# Self-assembly of plant protein fibrils interacting with superparamagnetic iron oxide nanoparticles

**DOI:** 10.1038/s41598-019-45437-z

**Published:** 2019-06-20

**Authors:** Jing Li, Ievgen Pylypchuk, Daniel P. Johansson, Vadim G. Kessler, Gulaim A. Seisenbaeva, Maud Langton

**Affiliations:** The Department of Molecular Sciences, SLU - Swedish University of Agricultural Sciences, Box 7015, SE-750 07 Uppsala, Sweden

**Keywords:** Scanning probe microscopy, Protein aggregation, Atomic force microscopy, Biomaterials - proteins, Chemical bonding

## Abstract

*In situ* fibrillation of plant proteins in presence of the superparamagnetic iron oxide nanoparticles (NP) promoted formation of a hybrid nanocomposite. The morphology of NP-fibril composite was revealed using *ex-situ* atomic force microscopy (AFM) in air. The NP-fibrils were associated into extended multi-fibril structures, indicating that the addition of NPs promoted protein association via β-sheet assembly. Real-time movement of NPs attached to fibrils under an external magnetic field was visualized using *in-situ* AFM in liquid, revealing that composite structures were stable at low pH, and displaying dipolar property of the NPs in the composite at high pH. Changes in magnetic properties of NPs when interacting with protein fibrils were quantitatively mapped using magnetic force microscopy (MFM). The magnetic moment of the NPs in composite was increased by co-existing with protein at low pH, while their dipolar nature was maintained at high pH. Self-assembly of the protein into fibrils is accelerated with increasing NP concentration within an optimal range, which is attributed to a fibrillation-competent conformation of the peptides. The latter was explained by the formation of favorable hydrogen bonds, electrostatic interactions, and efficient surface energy transfer between NPs and proteins.

## Introduction

Proteins display a strong propensity to adsorb onto surfaces via electrostatic interactions, coordinative bonds, hydrogen bonds, and hydrophobic interactions. When nanoparticles (NPs) come into contact with proteins in biological fluids, they will be immediately entrapped by those proteins. The establishment of this protein cloak, named protein corona, is a competitive process governed by the concentration of proximal free proteins, and the surface chemistry of NPs such as surface charge distribution, surfactant and chemical reactivity, NP size relative to that of the adsorbing proteins^1^. Newly designed magnetic NPs has been proposed to have potential usages within diagnostics of Alzheimer’s disease (AD)^2^. A few reviews emerged focusing on the study of different mechanisms of protein corona during protein-nanoparticle (NP) interactions e.g. how the magnetic iron oxide NPs influences protein conformation^3,4^.

Protein fibrils are self-assembled aggregates that can be produced through fibrillation of various types of protein at high temperature via protein unfolding followed by refolding of the protein into misfolded state, nucleation, and fiber elongation^5^. The field has attracted much scientific attention in conjunction with AD^6^. NPs present enormous surface areas and some of those are found acting as catalysts to enhance the rate of protein fibrillation by decreasing the lag time for nucleation of protein (β2-microglobulin) during the exchange of protein between solution and the NP surface. The mechanism of NP surface-assisted nucleation of proteins was attributed to the dependence of the concentration and nature of the NP surface^7^. This study provided insights on the controlled self-assembly of proteins into novel nanomaterials by further understanding the mechanism of nucleation kinetics between protein and NPs. Indeed, there has been growing interest in the study of amyloid-like fibril assembly within the fields of materials science, owing to advanced functionalities related to their structural polymorphism and specific physicochemical properties^8^.

Due to focus on mechanism of protein fibrillation, to date animal based β-lactoglobulin is among the most heavily investigated fibril forming proteins. The mechanism of self-assembly and fibrillation kinetics of β-lactoglobulin has been systematically investigated^9^. Growth kinetics of protein fibrils can be represented by a simple polymerization model including a hydrolysis step^10^. Development of functional composite nanomaterials using biomass resources such as plant-based raw materials is of importance globally to reach a more sustainable society. It is known that plant protein-based fibrils can be easily formed during heating at low pH and at a temperature of 80–85 °C. Studies of fibril formation from 7 S globulins extracted from soy and pea showed that β-sheet aggregates appeared due to conformational changes during the self-assembly of protein following the hydrolysis step at pH 2.0^11,12^. The generally accepted mechanism of globular protein conversion into amyloid fibrils includes multiple steps, from a partially unfolded conformation, proceeding through proto-fibrillar structures (oligomers and protofilaments) into mature fibrils. The first step is the assembly of oligomers from either unfolded protein that refolds into a cross-β-sheet or from short fragments (also folded into cross-β-sheets) created by hydrolysis of the monomeric form of the protein^13^. When protein is maintained at high temperature and low pH, these hydrolyzed fragments contribute to protein fibrillation. The energy consumed during fibrillation is spent on unfolding-refolding or unfolding-hydrolyzing the protein^14^. In a recent study on the role of peptide hydrolysis in bovine whey protein β-lactoglobulin fibrillation kinetics, we demonstrated that the balance between protein concentration and hydrolysis rate determined the structure of the amyloid fibrils formed^15^.

Studies on magnetic responsive composite materials using β-lactoglobulin-based amyloid fibrils and iron oxide NPs (Fe_3_O_4_) have provided great promise in design of functional colloidal systems, in which the aggregation behavior, orientational order of the composite were efficiently controlled in a purely noninvasive way by moderate magnetic fields of weak intensity^16,17^ Tuning of NP biological functionality can be achieved through controlled surface chemistry by manipulating the surface properties of the NPs at the bioconjugated interface between NP and protein surface^18^. It has been shown that different acidic coating decorated superparamagnetic iron oxide NPs (denoted as iron oxide NPs from now on) strongly influenced the protein (in plasma) corona composition and structure^19^. A study on effect of surface charge of dextran coating layer of superparamagnetic iron oxide NPs on the kinetics of fibrillation of amyloid-β (Aβ) in aqueous solution demonstrated a size and surface area dependent “dual” effect of the NPs on Aβ fibrillation^20^.

Of profound importance for study on the mechanism for understanding of iron oxide NP/fibril composite materials, method design for characterizing the orientation of covalently conjugated proteins/superparamagnetic NPs still remains a challenge. Even though extensive physicochemical characterization of targeting NPs can be addressed in detail, relevant quantitative biological characterization for real time study of the nano-interface is however challenging for selecting suitable nanomaterials for further *in vitro* or *in vivo* experiments^21^.

Still, little attention has been paid on quantitative evaluation of the relation between structure of the NP-fibril and change of the magnetic moments of the iron oxide NPs before and after interacting with fibrils. Further, to gain a microscopic molecular description of the biological identity of surface modified NP/protein fibrils, there has been a lack of attempt seeking for *in-situ* real time methods for gaining in-depth understanding of these processes of protein fibrillation when interacting with NPs in liquid, in order to better clarify the presentation of functional biomolecular motifs at its interface, and to identify the spatial location of proteins, their functional motifs and their binding sites^22^. Hence, this points out an urgent need for a smart design of methodology in this rapidly developing and not yet fully explored field.

The microstructure and fibrillar assembly kinetics of heat-induced fibrils and composite have been characterized by others using a variety of techniques and methods^23^. In particular, advanced AFM techniques including quantitative nanomechanical mapping have been used for studying the nanomechanical and aggregation mechanism of amyloid fibril materials^24,25^. Magnetic force microscopy (MFM) is a high spatially resolved, quantitative imaging technique that has been used extensively in research to detect probe-sample interactions, force gradient, and energy dissipation from superparamagnetic iron oxide NPs for applications such as biomedical devices^26,27^. Due to the appealing advantages of *in-situ* atomic force microscopy (AFM)- based methods, it thus appeared interesting to study the formation in real time in solution using *in-situ* AFM-based methods as a model platform for the studies of the self- assembly mechanism of NP-fibril structured functionalized materials^28^.

Yet few studies have focused on protein fibrillation using plant-based proteins. In this work we sought to provide a proof-of-principle for the potential role of magnetic nanoparticles in this process, using a simple and robust experimental setup. The detailed study of the action of magnetic field was though not set as an aim in this work. Oleate (surfactant)-modified iron oxide NPs have been studied by preparing dispersions of NPs with narrow size distribution (e.g., 10–20 nm) to obtain improved biocompatibility using different methods^29,30^. Though, formation of composite of plant protein fibrils and oleate surface-modified biocompatible Fe_3_O_4_ NPs, and use of MFM combined with *in-situ* AFM-based techniques for real-time study has not yet been reported to the best of our knowledge. It is of great interest to gain a better understanding of how those surfactants used to stabilize iron oxide NPs affect the mechanism of plant protein fibril formation, and how the correspondingly rendered surface properties and concentration of NPs can affect magnetic moments of the NPs during the process of plant protein fibrillation. The mechanisms to be demonstrated could be of importance in development of functional protein fibril-based composite materials in a controllable context.

**Figure 1:**
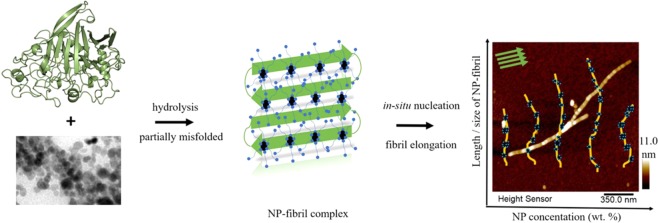
Schematic illustration of the proposed mechanism of *in situ* fibrillation of the protein in the presence of surface-modified NPs. The AFM image shows a zoomed-in view obtained in air of the representative morphology of a single NP-fibril composite. The protein β-sheet structure is illustrated schematically, where the AFM image shows a zoomed-in view of the representative morphology of a single NP-protein-fibril composite, displaying clear proof of the proposed mechanism.

As outlined in Fig. 1, in this study, we have investigated self-assembly mechanism of plant protein fibrils by introduction of the surface modified iron oxide (Fe_3_O_4_) NPs using oleate. The scheme displays that the length of fibril-NP composite showed dependency on concentration of NPs, and NP helped with accelerating the fibril formation within an optimal range, which can be due to formation of favorable hydrogen bonds, electrostatic interactions, and efficient surface energy transfer between NPs and proteins during *in situ* protein fibrillation.

Specific objectives of the study were: 1) to investigate formation of a hybrid composite of plant proteins and NPs in the conditions common for fibrillation; 2) to apply *in-situ* AFM/quantitative MFM methods to obtain fundamental understanding of the mechanism of fibril self-assembly in presence of the superparamagnetic oleate modified Fe_3_O_4_ NPs at low pH; 3) to identify challenges for development of functional fibril composite nano-/micromaterials. The design of our study for scalable framework structured protein-NP bio materials has demonstrated clear applicability of this for studies of different type of proteins used in various biological systems, and will be attractive to use within a range of applications.

## Results

### *Ex-situ* AFM in air showed size dependence of length of NP-fibril

**Figure 2:**
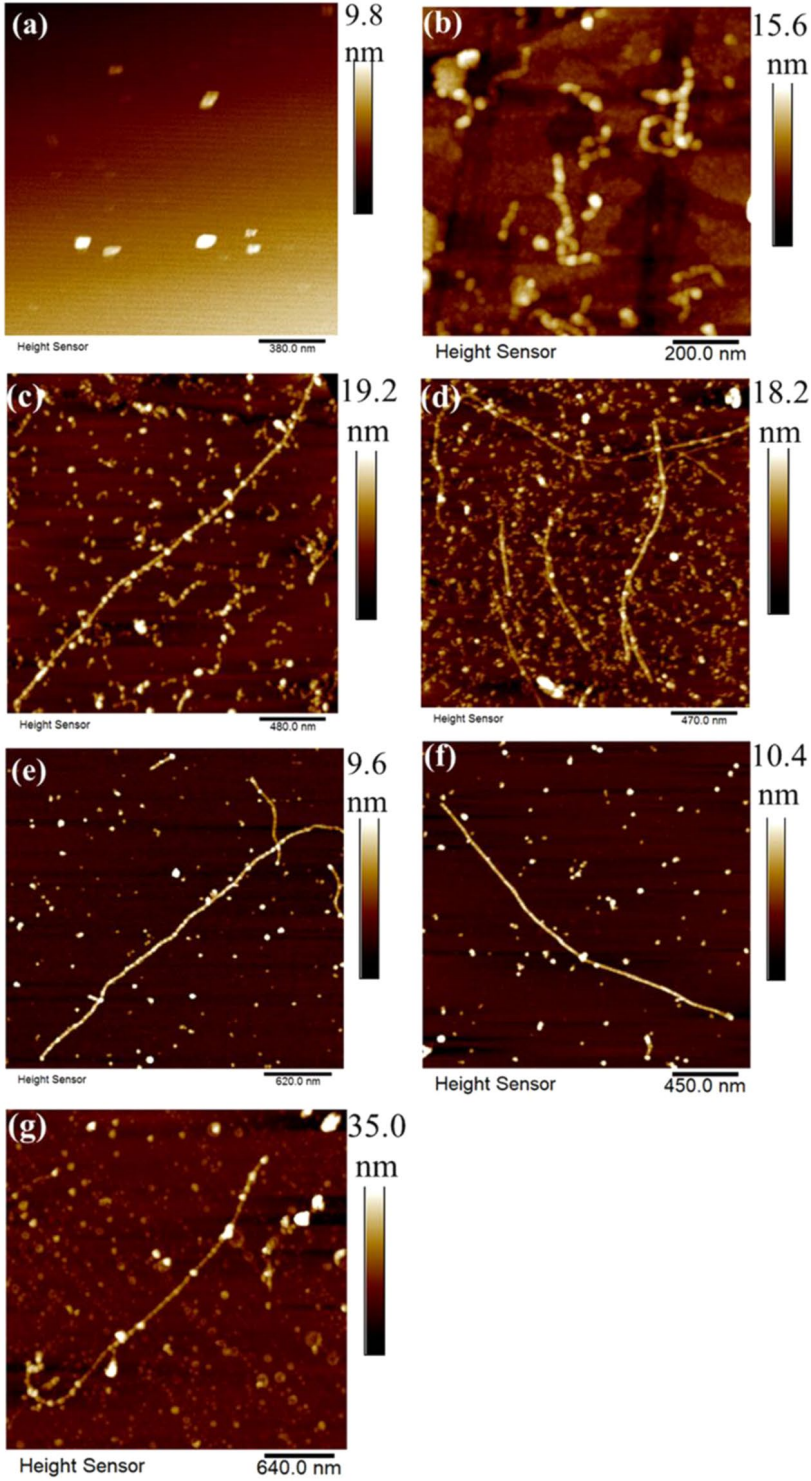
Typical AFM images obtained in air of (**a**) NPs, (**b**) fibrils, and (**c–g**) NP-fibril composite with different concentrations of Fe_3_O_4_ NPs: (**c**) 0.0003 wt.-%; (**d**) 0.0015 wt.-%; (**e**) 0.003 wt.-%; (**f**) 0.03 wt.-%; (**g**) 0. 3 wt.-%

AFM images of pure NPs (Fig. 2a), and mixtures with proteins at various NP concentrations (Fig. 2c–g) revealed varying combinations of curly short and long straight fibril morphology. The fibril sample prepared without NPs contained curly structures of length 200 ± 100.0 nm (Fig. 2b). The average pitch was 39.3 ± 14.2 nm. In the NP-fibril composite material (Fig. 2c–g), the uniformly distributed NPs were compactly attached onto long fibrils, up to microns in length. Most of the NPs were embedded in the long fibrils (Fig. 2c). The size distribution of fibrils narrowed and fibril length increased significantly with increasing NP concentration in the mixture, for a given incubation time. Average length of the fibrils formed with 0.03 wt.-% NPs contained in the protein water solution was 99-fold greater (max. ~5 µm) than that of fibrils (~50–100 nm) formed with the lowest concentration of NPs. A decreasing proportion of curly short fibrils may suggest an increase in protein fibrillation efficiency with increasing NP concentration.

**Figure 3:**
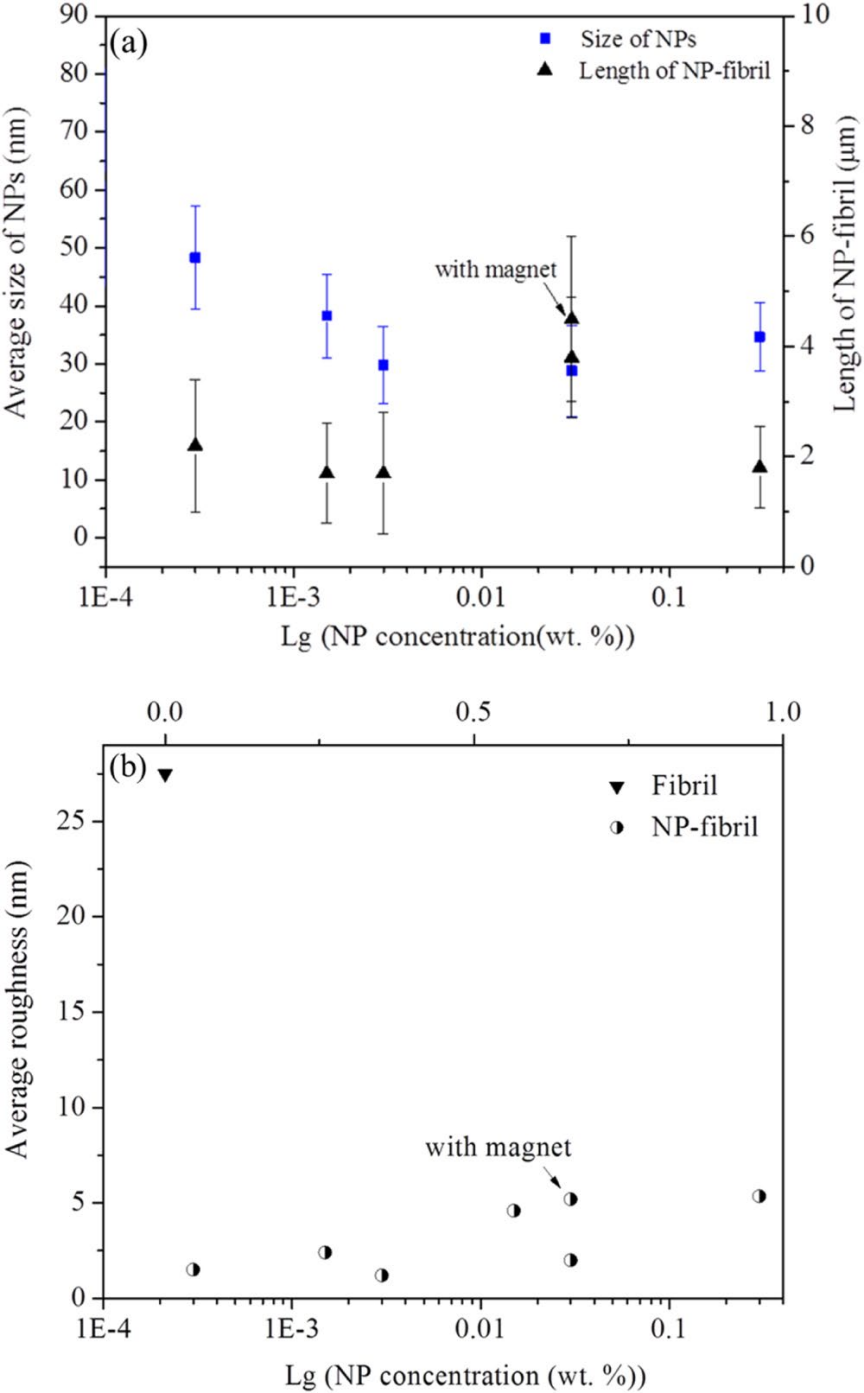
(**a**) Size of NPs and length of NP-fibril composite as a function of NP concentration. (**b**) Average roughness (R_a_) values as function of NP concentration.

Figure 3(a) illustrates the decrease in NP size as a function of NP concentration in the water solution. Statistical analysis revealed that the average size of NPs in composite samples was 48.3 ± 8.9, 38.3 ± 7.2, 29.8 ± 6.7, 28.8 ± 7.8, and 34.7 ± 5.9 nm at a NP concentration of 0.0003, 0.0015, 0.003, 0.03, and 0.3 wt.-%, respectively. Thus, NP size in the mixture decreased and then increased again with increased concentration of NPs in protein. Interestingly, roughness mapping of the AFM images, as indicated by the average roughness (R_a_) values in Fig. 3(b), showed that the overall roughness of the surface of the NP-fibril composite was significantly lower than that of fibrils obtained in the absence of NPs. This could be related to a decrease in the amount/proportion of shorter fibrils with increasing NP concentration. Moreover, as indicated by the arrows in Fig. 3, the size of NP and average roughness of the composite with 0.03 wt.-% NPs were both increased when measured with a magnet field applied comparing to the corresponding values obtained without magnet field. The results demonstrated that the magnetic properties of NPs in the composite changed under the applied magnetic field.

The *ex-situ* AFM images, statistical analysis of the size of the NPs in composite, and roughness mapping quantitatively showed that the morphology, length, and proportions of short and long fibrils were dependent on the concentration of NPs. Furthermore, the *ex-situ* AFM results in air indicated that NP concentration affected protein fibrillation, suggesting that physiochemical properties of the NPs, such as surface modification and surface charge, may have significantly influenced the self-assembly kinetics of the protein during *in situ* protein fibrillation. It is therefore likely that the hydrophilic oleate tail bonded to the surface of the NPs played an important role in *in situ* growth of protein fibrils during heating. It was concluded in a previous review that dissolved iron (Fe) often forms a hexahydrated ion, and that the reactivity of this depends on oxidation state and pH. These hydroxylated metal complexes are unstable in solution and contribute to growth of either iron oxide or hydroxide NPs, depending on the reaction conditions^31^. The size dependence of the NPs in NP-protein-fibril composite on NP concentration observed in this study when interacted with protein may be related to the Ostwald ripening principle. With increasing NP concentration, the NPs in the composite samples at a given incubation (fibrillation) time may have dissolved as the result of consumption of the hexahydrated ion from the surface at low pH during the ripening process, leading to decreasing NP size with an initial increase in NP concentration^32^. A tendency for iron crystal growth may demonstrate that the Ostwald ripening process could occur and contribute to formation of well-defined NP-protein-fibril composite under the experimental conditions used in this work. However, according to the AFM results a further increase in NP concentration did not favor continued growth of monodisperse NPs, but rather resulted in formation of large clusters of NPs. This observation may be related to the instability of surface energy of different facets of the particle itself, e.g., increasing NP concentration could lead to re-dissolution of smaller NPs with high energy facets and enhanced growth of other NPs in solution via intraparticle diffusion^31,33^. Further studies would be needed to confirm the exact mechanism of size dependence in nanocomposite formation.

### *In-situ* AFM in liquid with an external magnetic field visualized stability and change of dipolar properties of NP-fibril composite

**Figure 4:**
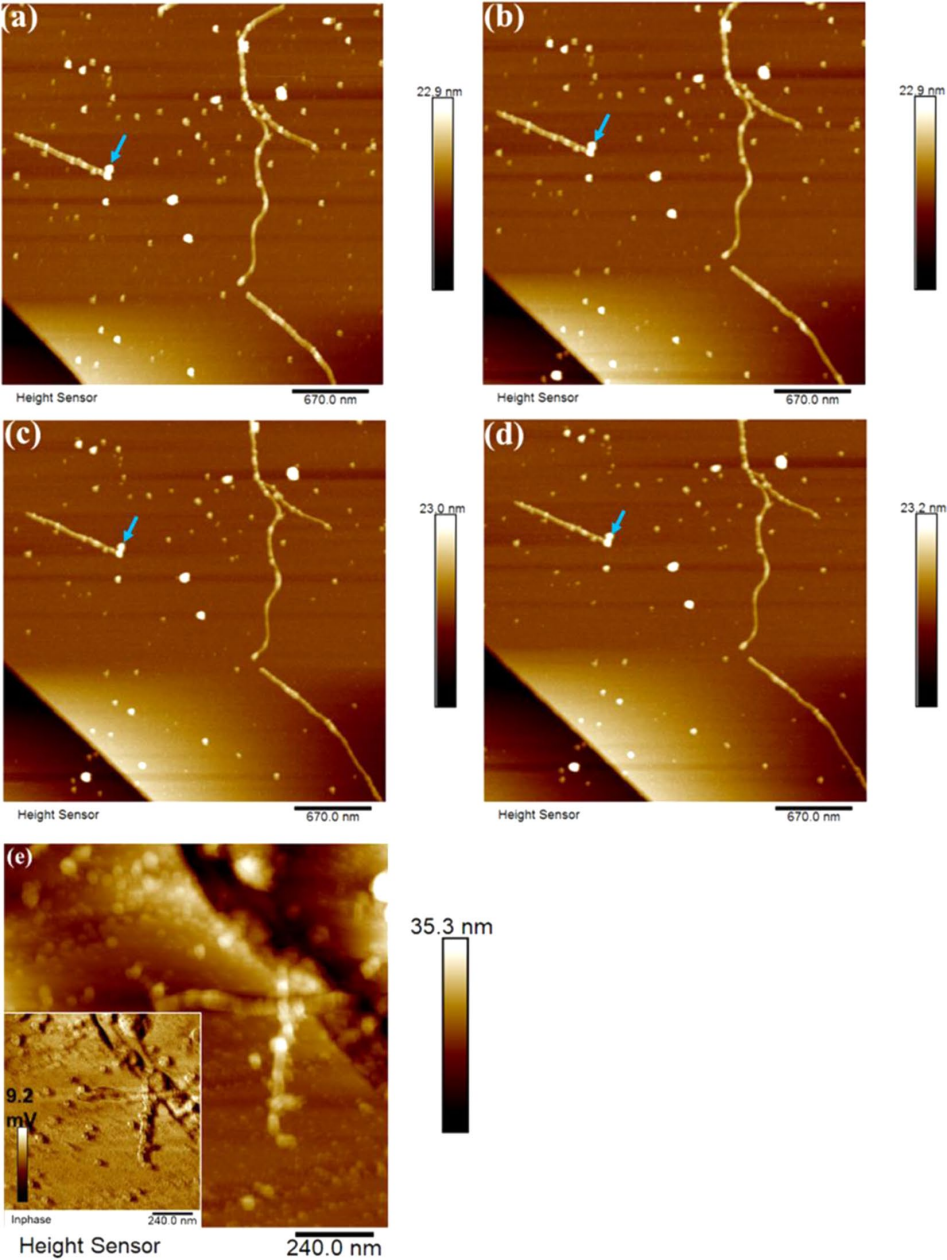
(**a,d**) Sequential representative AFM images of the nanoparticle (NP, 0.003 wt.-%)-fibril composite obtained under an external magnetic field over the course of measurement for 128 mins in liquid (**a**) before magnet; (**b**) 30 mins; (**c**) 72 mins; (**d**) 128 mins. (**e**) AFM morphology and in-phase (insert) images of a zoomed-in view of another area of the same sample obtained in liquid.

The sequential AFM images obtained over the time course of about two hours of measurement in water solution at pH 2.0 are illustrated in Fig. 4(a–d). The most striking observation from the images was that the NPs were indeed embedded and kept stabilized inside both long and short protein fibrils, even when under an external magnetic field, which supports the AFM results obtained in air. This indicates that the magnetic properties of the NPs were well maintained after bonding with the protein during fibrillation. An in-phase image of a zoomed-in area of the same sample, as shown in insert of Fig. 4(e), confirmed the stability of the NP imbedded in the fibrils.

**Figure 5:**
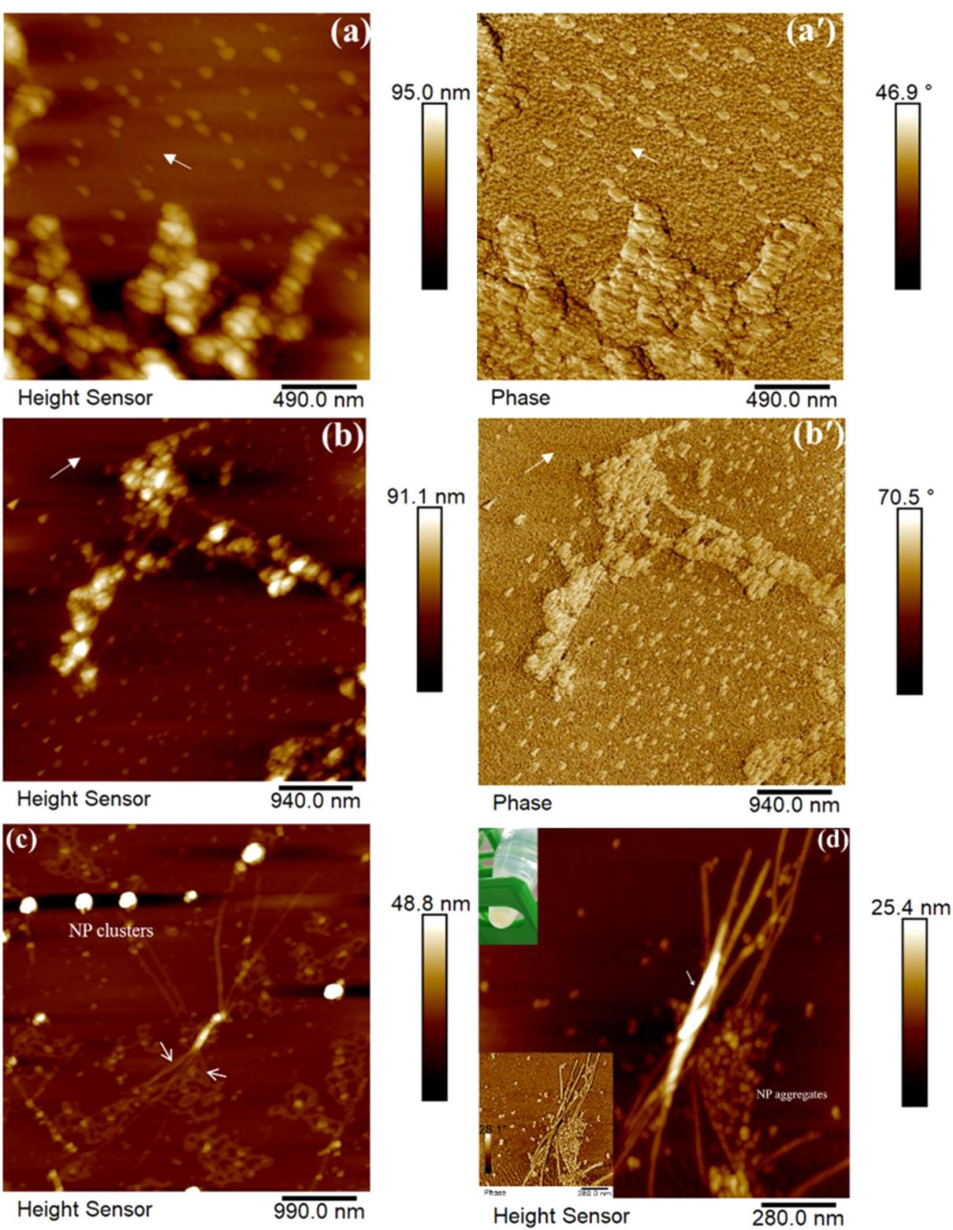
(**a**,**a**′); (**b**,**b**′) Representative AFM images and corresponding phase images of the nanoparticle (NP, 0.03 wt.-%)-fibril composite, obtained by scanning at two different locations on the same sample surface after adjusting the pH of the NP-fibril solution to 8.5. (**c**), (**d**) Typical AFM images of the sample obtained after one week of aging in solution under an external magnetic field. Insert: (upper) typical image of the sample solution after one week of aging; (lower) phase image of (**d**).

Response of the dipolar interactions of NPs co-existing with protein fibrils were visualized for composite samples by adjusting the pH of the NP-protein solution to 8.5, to ensure that the fibrils were deformed while the original shape of NPs was still maintained. This can be seen from the AFM images in Fig. 5((a,a′), (b,b′)), which were obtained by imaging two different locations on the surface of the same sample. As expected, the NP-fibril composite was deformed into shortish/round fibril aggregates. The typical triangular shape of NPs was maintained, which suggests that the surface modified NPs were stable even after deformation of the fibrils.

Most strikingly, as indicated with the arrows in Fig. 5, the orientational order of deformed fibril composite was in opposing directions (directing to left and right), reflecting dipolar interactions with NPs. This observation is well in agreement with previous obtained results^34,35^. Maghemite nano-cubic superlattices with a high degree of crystallographic orientational order can be produced and monitored by a magnetic field-induced nucleation and a self-assembly growth process. Using the magnetic field obtained from a normal magnet can induce a translational and orientational order of a superlattice of superparamagnetic maghemite nanocrystals, by temporal modulation of the dipolar interaction force^34^. As a result, the NPs inside fibrils may move towards each other.

The effect of long incubation times was investigated, by placing a normal magnet at the bottom of the solution of the as-prepared sample for one week. As shown in Fig. 5(c,d), empty tubes (as marked by arrows) were found inside the fibrils. Round NP clusters (large bright features in Fig. 5(c)) were formed afterwards at the end of the long fibrils or by attaching on the wall of long fibrils (marked by arrow in Fig. 5(d), with phase images shown as an insert at the left bottom), indicating that the magnetic NPs self-assembled by moving forward into each other when activated by the magnet. It may suggest that the orientational order of the NPs was altered under the magnetic field. An explanation can be that the particles move inside the fibers and also, when orientated by the field, contribute to formation of bunches of fibers. In this case, the round aggregates of “free” particles might originate through self-assembly of NPs not originally involved into formation of fibers.

Yellowish gel-like precipitations (phase separation) appeared at the bottom of the microcentrifuge tube, as shown in the insert in Fig. 5(d)), which illustrates that the fibrils get strongly aggregated in this case. This is in agreement with the roughness mapping as shown in Fig. 3, indicating that the NPs could be used to capture peptides and proteins in dilute and complex sample solutions. The surface of the NPs was modified using oleate, which contains a long carbon chain and an anionic carboxylate group, so the modified NPs can attract positively charged proteins through electrostatic attraction and hydrophilic-hydrophobic interaction.

The *in-situ* AFM results obtained in liquid with an external magnetic field applied demonstrated a high stability of the composite at low pH and well-maintained magnetic properties of the NPs in acidic solution. The observation indicates strong interaction and good compatibility of the NPs with the plant protein material used in the experiment. Our results also show the ability of the oleate modified NPs to act as affinity probes for study of charged species in protein-fibril materials. This is demonstrated by that a normal magnet can isolate NP-protein-fibrils in a solution fairly quickly, suggesting potential applications of this kind of composite material for e.g. magnetic imaging^30^.

### Changes in magnetic moment of the NP-fibril composite determined by quantitative MFM mapping

**Figure 6:**
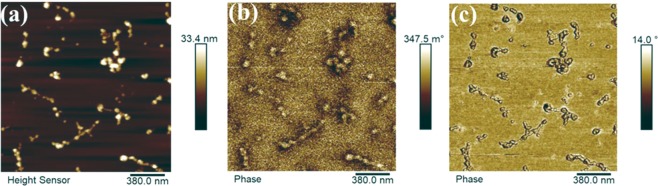
Representative MFM images mapped in tapping-mode (**a**) height, (**b**) phase shift, and (**c**) phase images of Fe_3_O_4_ NPs obtained in lift mode.

Figure 6(a,b) show the MFM morphological (height) image and phase shift image of the NPs. The average diameter of the particles was about 45 nm, measured using the height image. In phase images at 20 nm lift height when mapping with MFM, the NPs appeared as globular aggregates and bead-and-necklace like nanostructures with a positive phase contrast, which is associated with the phase shift of cantilever oscillations as a result of the interaction of the MFM tip with superparamagnetic polarized magnetic NPs. The phase shift due to background interaction is obtained as the average phase shift observed at a given lift height when using the MFM probe. The calculated phase shift for small and large pure NPs was 0.03245 ± 0.01055 and 0.0699 ± 0.005303 degrees, respectively. This indicates that the magnetic property of the NPs may be size-dependent. The bright features in Fig. 6 are probably NP clusters consisting of a few single NPs, judging from the phase image in Fig. 6(c).

**Figure 7:**
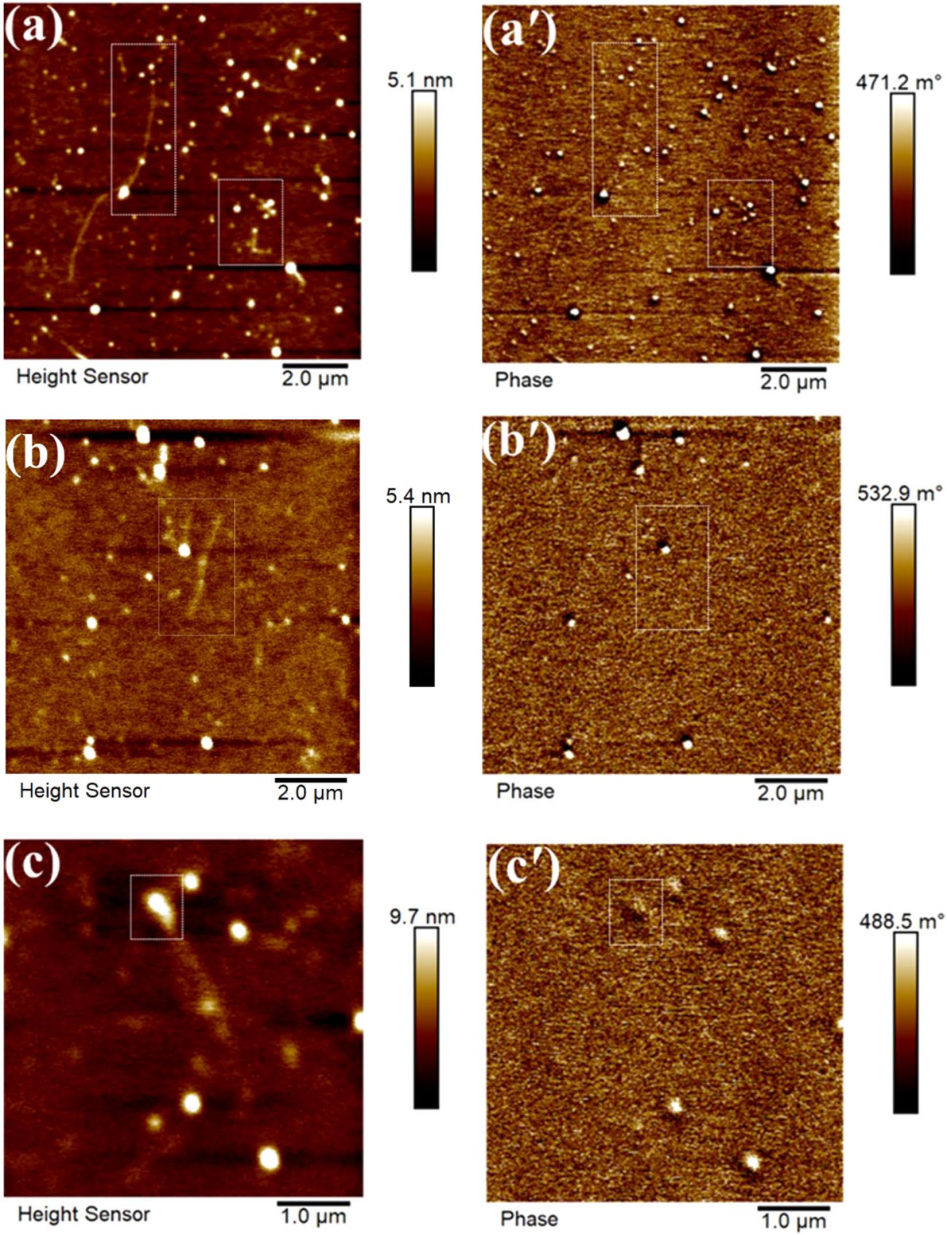
Representative MFM tapping mode height and phase shift images obtained in lift mode of three different locations of NP-fibril composite samples. (**a**,**a**′) area 1; (**b**,**b**′) area2; (**c**,**c**′) area 3.

For NP-fibril composite, the MFM morphological (height) images are shown in Fig. 7(a–c) and the phase shift images of the sample are shown in Fig. 7(a′–c′). As can be seen from the bright globular NP features attached internally and externally in the axial direction of the long fibrils, the smaller particles (bright features) likely embedded in the fibrils showed an average phase shift of a fraction of a degree, 0.1955 ± 0.0085. The large NPs (the brightest features) attached to the fibrils at the position marked by the arrows showed an average phase shift of 0.663 ± 0.391 degree.

In order to further verify whether the large Fe_3_O_4_ NPs were embedded in the fibril, MFM scanning was further performed for zoomed-in areas, as shown in Fig. 7(b,b′) and (c,c′). As expected, the phase shift information on the NP features in fibrils, represented as bright round clusters in the morphology image in the left panels in Fig. 7, was evident in the phase shift images (marked with squares). More strikingly, the phase shift of the relatively small NPs that were clearly imprinted inside the curly fibrils (as marked with the squares in Fig. 7) was of the same order of magnitude as that in the pure NPs.

Compared with the phase shift values of the pure Fe_3_O_4_ NPs, it is clear that the phase contrast shift of NPs in NP-protein fibril composite was significantly increased. These results indicate that the interaction of protein and NPs during *in situ* fibrillation modified the surface properties of the NPs, such as magnetic properties. Generally, magnetic moment of iron oxide NPs increases with their grain size because of the direct size effect^36^. Therefore the significant increase in the phase contrast of the large NPs in the NP-protein-fibril composite compared with the small NPs may be expected. This is in agreement with the size dependence of the NPs in NP-protein-fibril composite as a function of NP concentration, as observed from the *ex-situ* AFM results.

**Figure 8:**
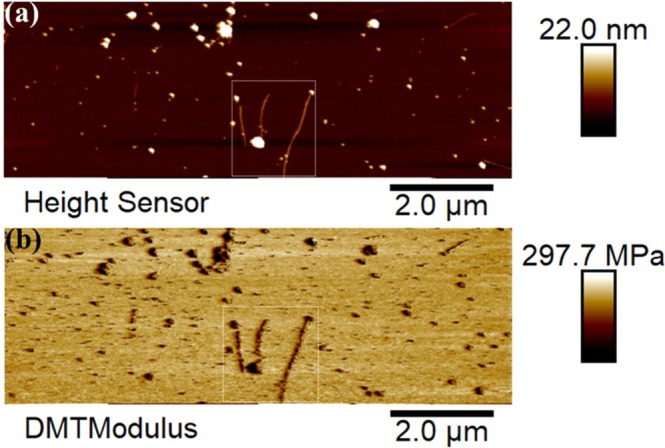
(**a**) Peak force tapping AFM morphology and (**b**) DMT modulus mapping of NP-protein-fibril composite, obtained in air using a magnetic probe.

In addition, the topographic mapping of the NP-protein-fibril composite revealed that their topography was less well defined than that of the pure NP as shown in Fig. 6. This can be caused by the presence of protein on the surface. It should be noted that the feedback mechanism in AFM tapping mode is to hold the oscillation amplitude constant. The amplitude depends on the tip-surface force in a non-linear way that cannot be completely defined. Thus, in order to obtain a clearer height image of the NP protein fibril composite, peak force tapping imaging was also performed for the same sample. As can be seen from the marked (square) features in Fig. 8, the NP-protein-fibril aggregates and the small NPs clearly attached on and imbedded in the long fibrils (as can be seen from the Derjaguin-Muller-Toporov (DMT) modulus map shown in Fig. 8(b)). This strongly supports the hypothesis that the phase shift response that was observed from the bright round features attached to/embedding in the long fibrils in Fig. 7 was due to a magnetic force arising from the magnetic moment of the NPs. This observation also demonstrates that AFM peak force tapping morphology mapping is beneficial in combination with MFM to generate complementary information on nanomaterials and their properties, due to the principle limitations of each technique.

The MFM results visualized the change in magnetic properties of the NPs when in composite with protein fibrils. The most unique observation is that the phase contrast shift of the NPs in NP-fibril composite was significantly increased when compared to that of pure NPs, which illustrated that the magnetic moments of the NPs can be real-time visualized by using MFM mapping. The magnetic properties of the NPs co-existing in composite with the protein fibrils displayed size dependence, which is agreement with our *ex-situ* AFM results. We can conclude that the magnetic properties of the NPs may be enhanced/or tailored by the protein interacting with the oleate tail during real-time growth of the NP-protein-fibril composite under the experimental conditions applied in this work.

As also shown by the topographic MFM images, in spite of that some of the very small Fe_3_O_4_ NPs embedded in the long protein-fibrils (as supported by SEM-EDS (results not shown) and peak force tapping AFM results in Fig. 8), did not show recognizable MFM phase contrast as observed from the phase mapping of the very small NPs. This may suggest that the superparamagnetic NP composition, with even smaller sizes as in this case, may become less magnetic during interaction with the protein during *in situ* fibrillation than when not bonded by the protein.

Previous studies have proposed that interactions of nitrogen group (NH-) and oxygen atoms of an organic surface modifier with sub-coordinated iron atoms on the NP surface may lead to a reduction in surface spin-canting disorder and may improve the spin rearrangement at the NP surface (thinner magnetic ′dead′ layers), as has been shown for magnetic NPs functionalized with oleic acid^37,38^. In the present study, this process could have been accelerated by interactions between the hydrolyzed protein fragments and the oleate, as discussed above. As a result, the magnetic phase shifts of the NPs, which attached on and clearly embedded in long and short fibrils, were increased. In addition, it has been reported that the original magnetic relaxivity of Fe (III) oleate NPs can be reduced by subsequent surface modification steps, such as ligand exchange processes^39^. Such reduction could not be completely ruled out in the present work, as there was no external magnetic field present during the MFM mapping. Nevertheless, the magnetic properties can still be sufficiently detected by MFM phase shift mapping, as shown in this work. Based on the results obtained, the plant protein/fibrils studied in this work are promising for modification of the magnetic properties of iron oxide nanomaterials in potential applications.

### Th T fluorescence spectra illustrated change of protein affinity following interaction with NPs

The advantage of using the Th T assay for studying changes in protein affinity is its simplicity. Because unbound Thioflavin T dye has fluorescence excitation (from 385 to 450 nm), enhanced fluorescence emission (from 445 to 482 nm) can be triggered and detected when the dye binds to protein β-sheet structures, resulting in a characteristic spectral shift^40^. This spectral shift can be used to differentiate bound and unbound Th T in order to reveal the presence of amyloid structures. In this study, Th T assay was used to analyze the change in amyloid formation of protein fibrils formed in the presence and absence of NPs. In order to confirm that there is no binding between the Th T dye and the NPs and that no signal of fluorescence of NPs is created under the experimental conditions, a Th T assay was also carried out for the pure NPs. As expected, negative values of Th T fluorescence (not shown here) were obtained for each of the pure NP sample tested, with the same NP concentration as was used for the composite samples, suggesting that no fluorescence was triggered by the NP at the excitation wavelength used in this work.

**Figure 9:**
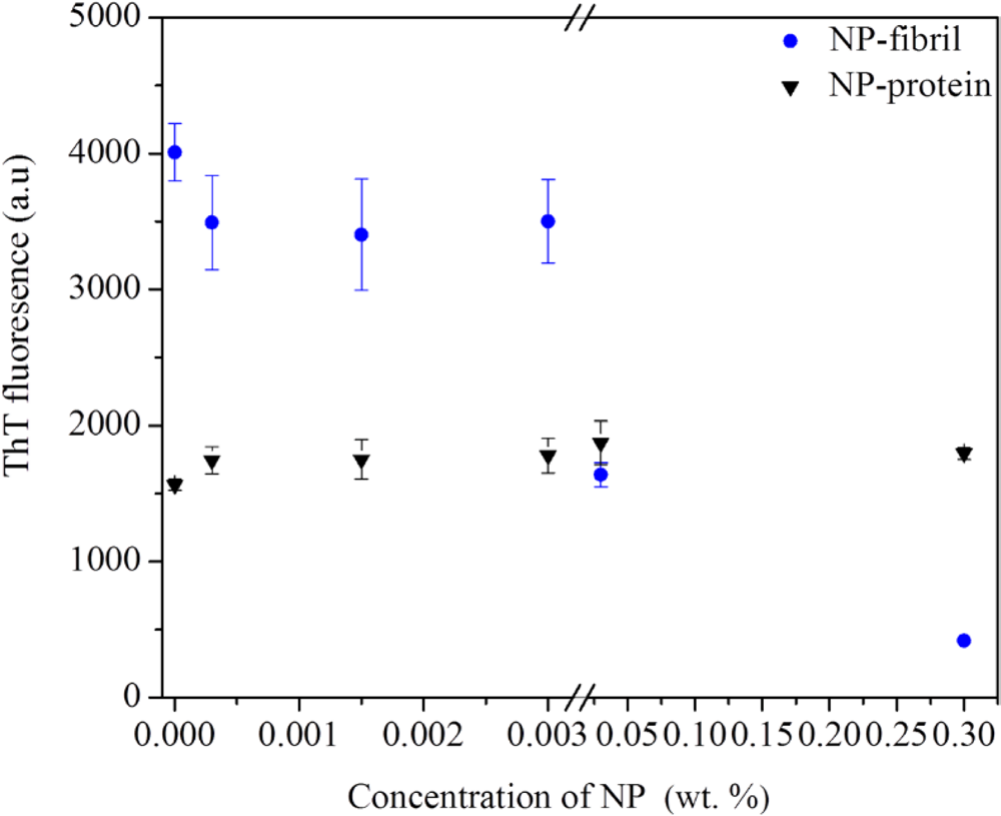
Thioflavin T (Th T) fluorescence spectrum of NP-fibril composite obtained in the absence and presence of NPs in acidic solution. Fluorescence spectra of native protein and pure NPs are shown for comparison (Dots in triangle).

As shown in Fig. 9, the fluorescence intensity of protein control sample (dots in triangle) slightly increased with increasing NP concentration, indicating that NP had a concentration-dependent effect on the ability of Th T dye to bind to the protein molecules. This could be attributed to negatively charged NPs effectively attaching onto positively charged areas of the protein monomers. More importantly, the fluorescence intensity of the NP-fibril composite samples was higher overall than that of control samples of protein and NP/protein without fibrillation, indicating that the amyloid structure appeared after heating the protein at low pH.

Moreover, the fluorescence intensity of the NP-fibril composite samples (dots in circles) increased overall with increasing NP concentration, indicating that NP also had a concentration-dependent effect on protein fibrillation. However, there was a sharp decrease when the concentration of NPs was higher than 0.003 wt.-%, suggesting that fibril formation may only increase when the amount of NP for protein fibrillation is in an optimal region. Fluorescence results obtained from another batch of protein sample as control, as shown in Fig. S3, clearly display a dramatic increase in fluorescence after fibrillation of the protein. This result is in good agreement with AFM-based results. These results indicate that addition of NPs facilitated the formation of fibrils, by acting as nucleation seeds during the formation lag phase and at early elongation of protein fibrils after hydrolysis of the monomeric proteins was completed. Thereafter, the NPs present at relative high density around the protein peptide were attracted to each other to enable *in situ* growth during fibrillation.

### Change in secondary structure conformation of the NP-protein-fibril composite was revealed by circular dichroism spectroscopy

**Figure 10:**
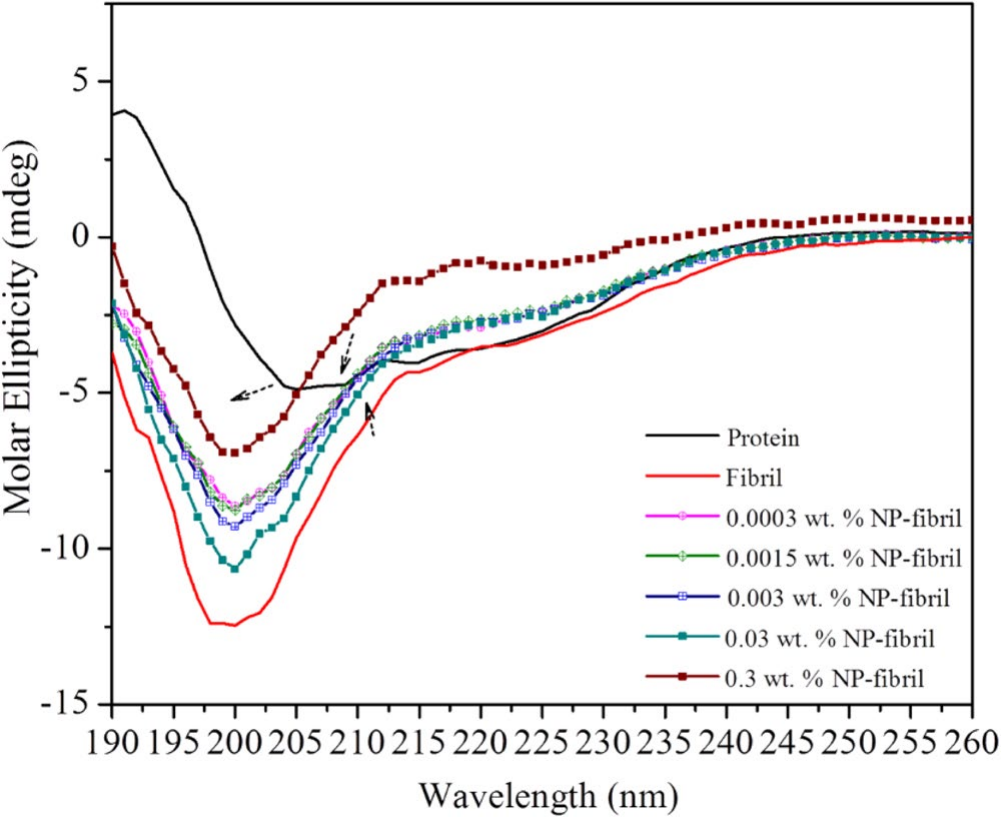
Representative circular dichroism spectroscopy (CD) spectra of nanoparticle (NP)-fibril composite samples obtained in the absence and presence of NPs in acidic solution. The spectrum of native protein is also shown for comparison (curve in black).

Far-ultraviolet (UV) circular dichroism (CD) spectroscopy was used to analyze changes in the conformation of the protein fibrils due to the interaction with Fe_3_O_4_ NPs at different NP concentrations. Figure 10 shows representative far-UV CD spectra of the protein fibrils without and with the presence of NPs. Generally, secondary structure elements of proteins, such as α-helices and β-sheets, have dichroic activity in the wavelength range 190–260 nm. In our CD results, the protein control samples (Fig. 10, curve in black) displayed two negative bands at 208 and 222 nm (indicated by dotted arrows), which are characteristic of α-helix structure in proteins^41^. The CD spectra of the fibril and NP-protein-fibril composite samples showed that a significant blue shift occurred for the negative band at 208 nm, which shifted to the band at 202 nm. The presence of β-rich aggregates in solution usually generates ellipticity minimum bands at ~215–220 nm and small values of millidegrees (mdeg)^42^. While the shift of the band indicates loss of α-helicity, with a concomitant increase in β-sheet structure, after heating at low pH^43^.

The spectrum of the control sample of protein fibrils exhibited far-UV spectra with prominent negative bands centered at around 202 nm, and a broad band at 210–215 nm. These bands suggest a highly ordered β-sheet structure of the protein fibrils, probably deriving from the β-sheet types that can be formed from the 7 S vicilin protein present in faba bean^44^. The shape and drift of the band at 202 nm for NP-protein-fibril composite samples illustrate that the presence and binding of NPs to protein led to significant decreases in the magnitude of the negative bands (less negative) of the spectra without an obvious shift in the peak position. This suggests an increase in β-sheet structure with the presence of NPs, which could be due to the change in protein secondary structure, i.e. a decrease in the α-helix content of the protein.

More importantly, the negative broad band centered at 210–215 nm in the presence of NPs became more recognizable (as indicated with the arrow in Fig. 10), which possibly reflects opening up of the compact transferrin conformation of the protein. This conformation can be related to thickness and surface charge of the oleate surface modifier of the NPs affecting the kinetics of protein fibrillation in aqueous solution, in agreement with previous findings^45,46^. This finding was also supported by the *ex-situ* AFM results showing the size-dependent nature of NPs in fibrils.

Moreover, the extent of decline in the same broad band increased with increasing NP concentration. Because increasing the NP concentration decreased the ratio of the protein to NP surface, the availability of NP per amino acid group on proteins significantly increased. As a result, more surface energy in the NPs could be quickly transferred to each protein molecule, causing stronger binding and leading to dramatic conformational changes between the NPs and protein^45,47^.

However, the extent of the decrease varied with NP concentration. For example, the spectrum for the composite sample obtained with the highest concentration of NPs (0.3 wt.-%) showed a decrease compared with the spectrum of the composite obtained with a concentration at 0.03 wt.-%. This can be due to the conformational changes of protein impeding the interaction between monomers of protein and NPs when the concentration of NPs was much higher than that of an assumed optimal region, further discussed in the next section.

### Polypeptide hydrolysis characterized by SDS-PAGE showed surface energy transfer between NP and protein fibril

**Figure 11:**
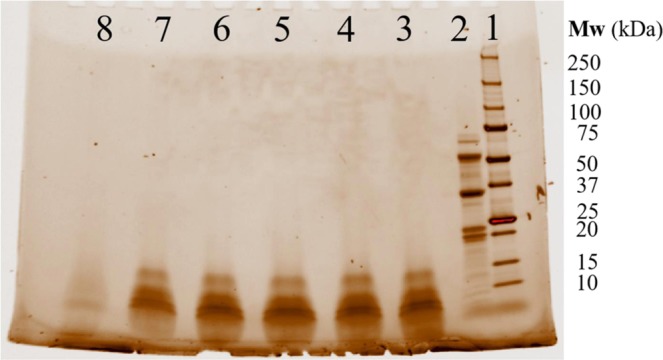
Representative SDS-PAGE results of protein, fibril and NP-fibril samples with different concentration of NPs. (lane 1), protein ladder; (lane 2), protein control; (lane 3) fibril control. (Lane 4–8) are results for NP-fibril samples. (lane 4), 0.0003 wt. % NP; (lane 5), 0.0015 wt. % NP; (lane 6), 0.003 wt. % NP; (lane 7), 0.03 wt. % NP; (lane 8), 0.3 wt. % NP.

The hypothesis that fibril growth was influenced by the binding energy between functional groups on protein and NPs surface during energy transfer in protein hydrolysis was investigated by SDS-PAGE gel electrophoresis. The characterization was applied to control samples of fibril and samples of NP-protein-fibril composite, where hydrolysis of the polypeptides of fibrils and NP-fibril composite was monitored using reducing SDS-PAGE. The major storage protein portions in faba bean are globulins, consisting of two high-molecular-weight proteins: legumin (11S) and vicilin (7S). Native legumin (11 S globulin) is an hexameric protein with molecular weight (M_W_) ranging from 330 to 410 kDa, consisting of one acidic α (M_W_: 35–43 kDa) polypeptide and one basic β (M_W_ 19–23 kDa) polypeptide bounded by a disulfide bridge. 7S vicilin/convicilin originates from proteolysis of a precursor (50 kDa) composed of three major subunits: α (20 kDa), β (13 kDa), and γ (12–16 kDa)^48^. As shown in Fig. 11, the protein control sample displayed typical polypeptide electrophoresis bands, among which 7S globulin consisted of α, α´, and β subunits with molecular weight of about 100, 70–75, and 50 kDa, respectively, and the 7S vicilin comprised a trimer of 50-kDa subunits, composed of 47, 27, and 22 kDa subunits, respectively. These results are in agreement with previous findings^49^. As expected, dramatic hydrolysis (i.e. proteolysis of protein) occurred for all the samples of fibrils and NP-fibril composite. For most NP-fibril composite samples, with increasing NP concentration, i.e., decreasing protein to NP surface ratio, the remaining proteins on the surface of NPs increased compared with the fibril (control) sample without NPs. This can be attributed to enhancement of the binding energy between protein and NPs during energy transfer during protein unfolding or proteolytic cleavage of the peptides^13,45^. We also found that the composite sample with the highest concentration of NPs (0.3 wt.-%) showed significantly different polypeptide cleavage behavior compared to samples with lower NP concentrations.

The Th T assay and SDS-PAGE results confirmed that the presence of NPs significantly contributed to formation and binding of hydrolyzed protein onto the NP surfaces, which was also demonstrated by the CD results. Where the introduction and increasing concentration of NPs resulted in a marked decrease in ellipticity magnitude (degree) in the negative bands, indicating a considerable increase in β-type secondary structure. This was possibly due to 7 S vicilin residues.

### Fourier transform infrared spectroscopy reflects the binding affinity of NP-fibril composite

**Figure 12:**
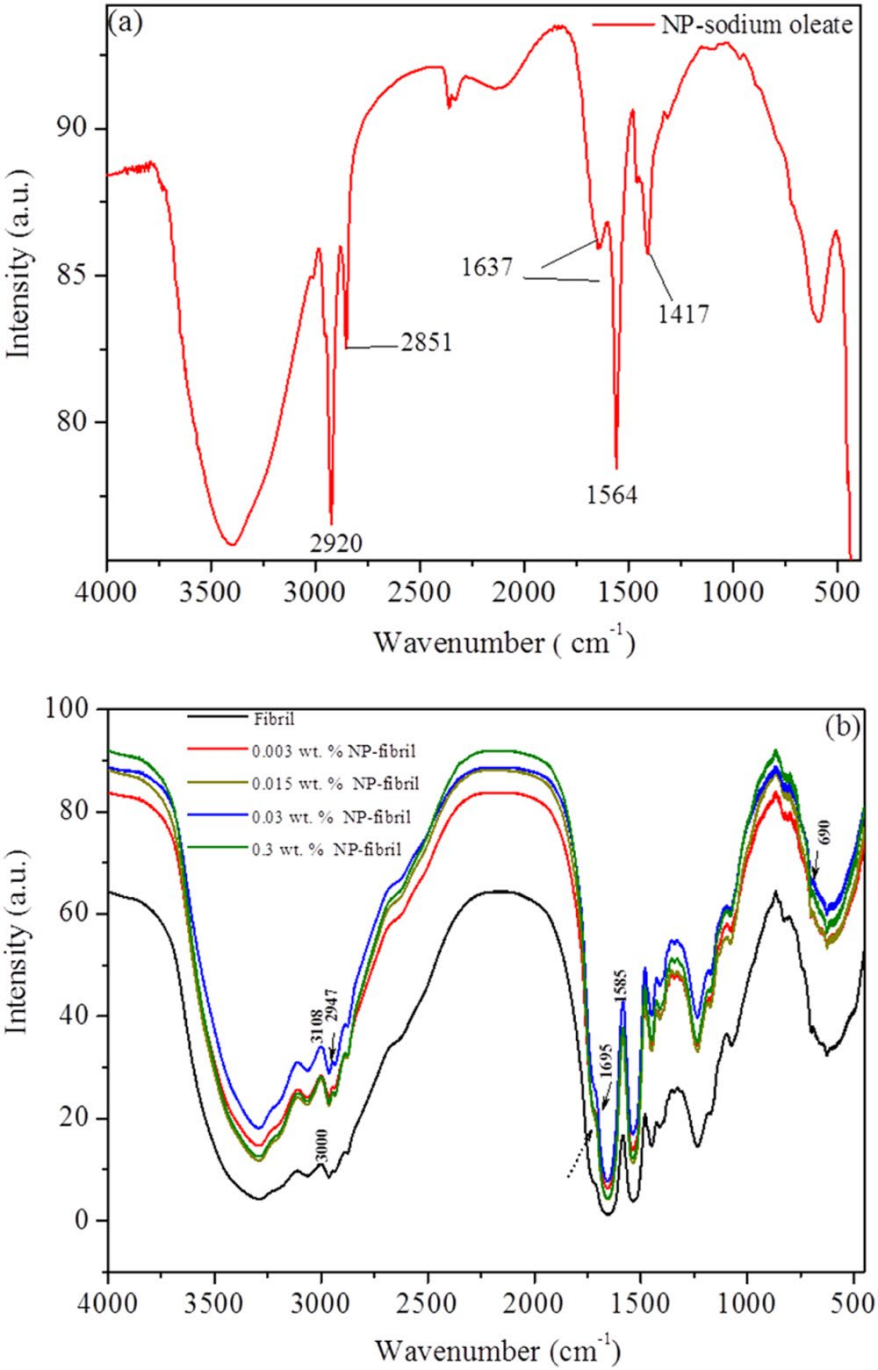
FTIR spectra of (**a**) as-prepared nanoparticle (NP) fluid and (**b**) protein fibrils and NP-protein-fibril composite with different concentration of NPs.

The Fourier transform infrared (FTIR) spectroscopy approach is a critical method for determination of protein secondary structure^50^. For the control sample of oleate-modified NPs in suspension (Fig. 12(a)), the broad FTIR adsorption band from 3600 to 3100 cm^−1^ corresponds to the stretching vibrations O-H of hydroxyl groups. The bands at 2920 and 2851 cm^−1^ can be assigned to asymmetric and symmetric CH_2_ stretch vibrations, respectively. The peak at 1637 and 1564 cm^−1^ is due to the presence of symmetric (ν_s_) and asymmetric (ν_as_) stretching vibrations of the –COO– group, respectively. The strong band at 1564 cm^−1^ indicates a non-coordinated –COO– group, due to excess sodium oleate in solution. The band at 1417 cm^−1^ can be attributed to –OH vibrations (Fig. 12(a))^51^.

Generally, the FTIR spectrum of domain structure bands of 7 S and 11 S globulin can be determined at 1600–1700, 1480–1575, 1220–1300, 3330, 1448, and 1395 cm^−1^^52^. FTIR spectroscopy has also been employed as a useful tool to reveal structural differences between native β-sheet proteins and amyloid fibrils^53^. However, to confirm the exact characteristic position of protein/fibril functional groups, one should be aware of that there are differences in shape and position of the amide maxima that can be displayed differently for samples prepared using different experimental conditions and systems, which can be related to that structural forms of residues of plant protein contain large amounts of extended conformation. The FTIR spectra of the protein fibrils at pH 2.0 in the absence and presence (curves in different colors) of the NPs at different concentrations are shown in Fig. 12(b).

As can be seen in the IR spectrum of the control sample of fibrils (black curve in Fig. 12(b)) show a peak at 1585 cm^−1^ and low-intensity peak of 1695 cm^−1^. These are assigned to the vibrational band of N–H and C=N stretching modes (amide II) and the stretching band of C=O (amide I), respectively, indicating the appearance of β-sheets. The appearance of the band at the very low wavenumber may imply that the formed fibrils adopt parallel β-sheet configuration^54^. The peaks located at 3000 and 3108 cm^−1^ correspond to the stretching vibration of N–H. The IR spectrum of the NP-fibril composite samples (Fig. 12(b)) was composed of strong and weak bands belonging both to the β-sheet structure of the fibrils (e.g., 1585 and 1695 cm^−1^)^55^, and the iron oxide NPs (690 and 2947 cm^−1^). Where the typical absorbance peak at 690 cm^−l^ can be assigned to the vibration band of Fe–O, the weak peak at 2947 cm^−1^ corresponds to the vibrational band of O–H. The latter peak is probably due to the presence of surface hydroxyl groups (Fe–OH) of the iron oxide and carboxyl groups of the oleate^56^. These results clearly illustrate attachment of the surface-modified NPs to the surface of the protein fibrils.

Blue shifts in the 1695 cm^−1^ band of the protein fibrils to a slightly lower band were observed for the spectra of NP-protein-fibril composite (as indicated by the arrow in Fig. 12), demonstrating structural transformation to an intermolecular hydrogen-bond between the β-strands in amyloid structure and NP surface^57^. The increase in intensity and expanse of the band (e.g., curve in blue for 0.03 wt.-%) can be associated with the increase in β-sheets and exposed β-strands^58^.

This suggests formation/existence of hydrogen bonding during the intercalation of protein molecules with the negatively charged NPs. Previous studies have indicated that the C–O– containing molecules and amide tend to form hydrogen bonds with the polar groups on the layered surfaces of the metallic NPs^59^. The –OH groups contained in the modifier on the surface of NPs could act as hydrogen binding sites for other bonded molecules^60^.

Since the isoelectric point of native 7 S globulin is approximately pH 4.4–4.8, the zeta potential of the protein fibrils is 37.8 mV (see Fig. S2). The protein surface will therefore be positively charges at pH 2.0^12,61,62^. Thus, by corroborating with zeta potential results for the negatively charged NP suspension, we can conclude that the heterogeneous distribution of the positive charges on the protein fibril surface probably enabled the positively charged amino acids on the surface of the protein to intercalate with the surface of NPs via surface electrostatic interaction and hydrogen bonding^63^. This result in a high affinity between the protein molecules and NPs.

However, it is worth noting that the highest concentration of NPs tested in this study (0.3 wt.-%) did not favor the formation of e.g. cross-β-sheets compared with lower concentrations. This is well in agreement with the SDS-PAGE results as discussed above, where the sample with the 0.3 wt.-% concentration of NPs displayed less NP attachment than samples with lower concentrations of NPs. Thus, although the ratio of NP layer per contacted protein molecules increased with increasing NP concentration, surface energy transfer from the NPs to the protein surface may have been impeded when the interaction between NPs and formation of NP aggregates began to compete with the interaction between NPs and protein peptides. This hypothesis is supported by the *ex-situ* AFM results for the composite sample obtained with the highest NP concentration, which revealed increased aggregation/large clusters of the NPs. These results thus suggest that there could have been an optimal concentration of NPs, i.e., an optimal protein to NP-surface ratio (e.g., 0.003–0.03 wt.-%), that facilitated protein fibrillation by building up an effective interaction through hydrogen bonding or/and surface electrostatic interaction, i.e., when the composite system reached the lowest Gibbs free energy than of the previous system with all reacting molecules involved^64^. The fibrillation kinetics of protein on NP surfaces could be described as fast equilibrium adsorption at the beginning of protein hydrolysis, followed by slow reversible unfolding of protein that facilitates adsorption of NPs by reducing the free energy barrier, when unfolding underwent hydrophobic interactions between the exposed protein residues and the hydrophilic surface of NPs^65^. Further studies on the kinetics of NP/protein *in situ* fibrillation are needed, using e.g., surface plasmon resonance, quartz crystal microbalance or *in situ* ellipsometry techniques^66,67^.

## Discussion

Taken as a whole, as illustrated in Fig. 1, our results clearly demonstrated that NPs were embedded inside the formed protein fibrils, and that NP-fibril assembly involved protein unfolding/hydrolysis/nucleation/elongation and fibrillation. Increasing NP concentration progressively increased the proportion of long fibrils formed. And as confirmed by the FTIR results, we could conclude that introduction of NPs significantly increased the formation of β-type secondary structure and self-assembly of the long protein fibrils, which was related to conformational changes.

Based on the results from our study, it can be hypothesized that during reaction of the negatively charged NP surfaces with the positively charged amino acid of the protein, the interaction was spontaneous and electrostatic interactions played a key role in the reaction process. The surface of the Fe_3_O_4_ NPs was disturbed by the change of microenvironment of amino acid residues, which may be positioned at the α β-strand interface of the globulin protein. The interaction of protein with NP surfaces results in conformational changes of the protein affecting the secondary and possibly tertiary structure of the protein, through changes of the van der Waal interactions, hydrogen bond interactions, and electrostatic interactions in the protein. As a result, the oleate tails on the NP surfaces may have bonded to the protein via hydrogen bonds during e.g., polypeptide hydrolysis, assembly, and entanglement of the protein fragments^68^. During this, removal of the crystal hydrate water and dissociation of oleic acid dimers could occur, so a more thermally stable iron-oleate complex could be formed with the protein fibrils when the fibrillation is completed^51^.

Negatively charged NPs can promote protein fibrillation at high particle concentrations, which can be attributed to changes in the ratio of NPs to monomeric proteins in solution and thereby the nucleation time, determining the binding affinity and kinetics of protein conformation^20^. Legume proteins with net positive charges at low pH, exhibit very high affinity to negatively charged NP layers. Specifically, in this study anionic superparamagnetic maghemite NPs showed a high affinity for biomolecules, an effect can mainly be attributed to electrostatic potential distribution on the protein surfaces. The self-assembly of protein can be improved by decreased intermolecular electrostatic repulsion interactions and increased molecular flexibility as a result of disruption of secondary conformations^69–72^. In the present case, the positive-charge-rich areas on the plant protein molecule surfaces were able to anchor the protein molecules into the negatively charged NP surfaces.

It has been reported that either the lag phase or the elongation rate of amyloid-β peptides (Aβ) fibrillation can be essentially affected in the presence of iron oxide NPs, as physiochemical properties of the NPs, depending on the function of the surface modifier, can change the aggregation kinetics of proteins^20^. When the NP concentration was lower than 0.003 wt.-%, the electrostatic interactions between the negatively charged NP surface and positively charged protein residues at low pH led to conformational changes of the protein, which could significantly decrease the nucleation lag time by making the altered protein conformation more prone to fibrillation. However, when the concentration of NPs increases too much, fibrillation of protein may be inhibited, as indicated by the sharp decrease in fluorescence for the fibril samples at an NP concentration higher than 0.003 wt.-%. This may be due to that the NPs at very high concentration modified the hydrophobic core of the β-sheet peptides and the backbone hydrogen bonding of the protein, which is responsible for self-recognition and assembly of protein fibrils, thus leading to strong inhibition of protein fibril formation either by impeding fibrillation or disassembling any fibrils that were formed^20,73^.

It has been proven that for globular protein such as β-lactoglobulin, the conformational changes are attributable to the transition between dimer-monomer and to rearrangement of the monomer structure itself, with consequent polymerization due essentially to the hydrophobic interactions^74^. Different native protein structures lead to different mechanisms in the protein aggregation process. The heating-induced aggregation pathway of the proteins can comprise a sequence of different steps in terms of changes in quaternary, tertiary, and secondary protein structures^74^. The conformational changes of the proteins result in exposure of specific amino acid residues, such as cysteine and hydrophobic regions, which play an important role in the whole process of protein aggregation. These changes can continue after exposure of hydrophobic areas of the protein, leading to the formation of very different protein structures at tertiary and even secondary level. Therefore, successive linear polymerization and a final rearrangement in macromolecular aggregates can be established^75^.

As shown in this study, and shown in previous studies, the introduction of surface-modified NPs into the protein solution may have strongly influenced the environment of hydrophilicity, as well as the structure of the protein peptides during hydrolysis. Thus, the exposed hydrophobic surfaces of the protein probably interacted actively with the oleate-modified hydrophilic NP surfaces. There was probably also larger surface accessibility of hydrogen bonding groups to the protein backbone during the change to the structured conformation under heating, resulting in that the NPs being attached onto the surface of the protein molecules^76^. Thereafter, the NPs can react *in situ* with the protein molecules by acting as seeds during nucleation and elongation of the protein peptides, leading to accelerated growth of long protein fibrils.

The mechanism of the interaction in self-assembly of the composite material can be explained as increasing concentration of NPs improving the rate of protein fibrillation by decreasing the lag time of nucleation^7^. The *in situ* fibrillation of NP-protein composite was therefore enabled by the simultaneous growth of fibril-forming peptides that effectively interacted with the NPs in steps of the reaction such as initial protein hydrolysis and *in situ* nucleation, therefore improving the self-assembly efficiency of fibrils. Protein peptide hydrolysis can be a rate-determining step for the formation of fibril-forming protein peptides in the subsequent steps^15^. The improvement of bioconjugation is probably associated with the negative charge and the bonds of carboxylic acid functional groups in the organic modifier (here, i.e. oleate) on the NP surfaces^77^. Our results may indicate increased efficiency of protein fibrillation in the presence of oleate-modified NPs within a certain optimal concentration range (i.e., 0.003–0.03 wt.-%), in which *in situ* fibrillation of the nanocomposite was facilitated by simultaneous growth of fibril-forming peptides that interacted with NPs close by. As a result, fibrillation-competent conformation of the peptides accelerated protein fibrillation in the presence of NPs.

## Conclusions

A novel structured Fe_3_O_4_ NP-protein fibril hybrid composite material was developed. *Ex-situ* AFM-based results revealed size dependence of NPs in composite on NP concentration. *In-situ* AFM and MFM results visualized the dynamic changes in structure and magnetic properties of the NP-protein-fibril composite. Combined Th T, CD and SDS-PAGE results indicated that secondary structure conformation, β-sheet formation, and self-assembly of the protein was accelerated with increasing concentration of oleate-modified Fe_3_O_4_ NPs, which acted as seeds during *in situ* growth of protein peptides to generate long fibrils. We conclude that protein binding at the surface of NPs in a fibrillation-competent conformation leads to improved formation of critical nuclei and fibrillation. This can be attributed to: (1) strong bonding of carboxylate groups of the oleate modifier onto the protein during hydrolysis; (2) electrostatic screening caused by active interaction of the negatively charged NP surfaces with positively charged surfaces/polar groups of the amino acids of the protein, such as e.g. cysteine, tyrosine, and lysine amino acids; and (3) efficient surface energy transfer between NP surfaces and exposed surfaces of protein residues as a result of the conformational changes of proteins during fibrillation. The speed of protein oligomer elongation increased with increasing ratio of NP surface area to protein surface area, which may give an optimal range of NP concentration. Our work is a simple and scalable platform for design of protein-NP structured framework system using plant proteins, which could be further developed to make production of a variety of amyloid fibril-based protein/bio-compatible NP functional materials. The results will be of profound interest for the development of potential applications in e.g. bio degradable materials.

## Methods

### AFM imaging of NP-fibril composite and dipolar interactions of NPs, in air and liquid with an external magnetic field

AFM imaging in air and liquid was performed in tapping mode using a Bruker Dimension Fast-scan instrument. Rectangular silicon nitride cantilevers (Bruker, FASTSCAN-A, 1400 kHz resonant frequency) with a nominal spring constant of ~18 N/m and a tip radius of 5 nm were employed for imaging under ambient conditions (air). Rectangular silicon nitride cantilevers (Bruker, SCANASYST-fluid + , 150 kHz resonant frequency) with a nominal spring constant of ~0.7 N/m and a tip radius of 2 nm were used for imaging in liquid. (Note that cantilevers were also individually measured for the amplitude versus frequency spectrum to obtain lock-in frequency) the spring constant and resonance frequency of each cantilever were determined more precisely for each cantilever used.) A 20 μL droplet of sample (i.e., NP, fibril, and NP-fibril composite) solutions (50-fold diluted) was spread on a freshly cleaved mica disc and dried in air using N_2_ flowing gas. Images were processed using Nanoscope Image Analysis Software (Version 1.7). The full preparation and imaging procedure was repeated three times to ensure reproducibility. Approximately 10–30 images were obtained for each sample. Samples for AFM imaging under an applied magnetic field were prepared using two small normal magnets (NdFeB block, 10 × 10 × 10 mm, (N48), Ni + Cu + Ni + Au (Gold) (8505110000) (Radiomag GMBH). Magnetic flux density −1360–1420 mT. Magnetic field strength −836 kA/m) mounted horizontally to the left and right of a drop of sample solution (i.e., 20 µL 0.03 wt.-% NP-fibril composite at pH = 8.5) on a cleaved mica surface and dried in air for 12 hours. Representative locations on the same sample were investigated.

### Visualization of magnetic properties of NP-fibril composite using MFM in air

MFM imaging was performed with a Bruker Dimension Icon using CoCr-coated etched silicon tips (MESP-V2, f_0_ = 75 kHz, length = 225 um). The magnetic coating consists of ~35 nm of Co/Cr alloy (exact thickness and composition of the coatings varies). The coating has a coercivity of ~400 Oe and a magnetic moment of 1×10^−13^ emu. In order to ensure a predominant orientation of the magnetic vector field along the major probe axis, the magnetic probes were first magnetized for about one second (along the cantilever) using a standard magnetizing device containing a permanent magnet. The apparatus ensures that the distance from the magnet to the tip is the same for each probe. Quantitative data on the stray field of iron oxide NPs can only be derived from MFM images after accounting for the topography. Therefore, in this work a lift mode was used, where the topography on each scan line was first measured with the tip in immediate proximity of the surface, after which the magnetic interaction was measured in a second scan of the same line at a constant lift height. The lift heights chosen in this work was 20 and 50 nm. Topography was collected in tapping mode, the data recorded to one image channel. The phase shift was used as contrast channel in lift mode to highlight variations in magnetic response, due to high signal-to-noise ratio. A drop (20 µL) of 0.3 wt.-% NP-fibril composite in aqueous suspension was quickly dried onto a mica surface by blowing with N_2_ gas before starting the measurement. A control sample of NPs deposited from aqueous suspension was imaged for comparison. It should be kept in mind that because MFM is sensitive to the strength and polarity of near-surface stray fields produced by ferromagnetic domains, rather than to the magnetization itself, it is not straightforward to deduce the overall domain topology from an MFM image. The statistical data was analyzed using Nanoscope analysis for flattening (1^st^ order) and calculation of magnetic phase shift and Gwyddion for statistical analysis^78^.

### Circular dichroism (CD) spectroscopy

Far-ultraviolet (UV) CD spectra were obtained using a JASCO (J-810–150S) spectropolarimeter. The measurements were performed in a quartz cuvette of 0.2 cm with different concentrations of the samples in 0.01 M/L HCl solution. Scanning was performed from 190 to 260 nm, with a scan rate of 50 nm per minute. Final spectra were an average of 20 scans. The spectra were recorded using the following parameters: step resolution 1 nm; acquisition duration 1 s; bandwidth 5.0 nm; sensitivity 100 mdeg. The cell was thermostatted with a Peltier element at 20 °C. Recorded spectra were corrected by subtraction of a background measured in 0.01 M/L HCl solution (pH = 2.0). Each data point was the mean of duplicate measurements.

### Thioflavin T (Th T) fluorescence and SDS-PAGE analysis

The Th T stock solution was prepared by dispersing 8 mg of Th T in 10 mL of phosphate buffered saline (pH 7.0) containing 150 mM NaCl and stored in darkness before starting the measurements. The stock solution was diluted 50-fold in the same buffer on the day of analysis to generate the working solution. Aliquots (20 μL) of the control sample of fibrils and of NP-fibril composite were mixed with 5 mL of Th T working solution and allowed to stand for at least 1 min. The fluorescence spectra of the mixtures were measured using a fluorescence spectrophotometer (POLAR star Omega). The excitation wavelength was 440 nm (slit width = 10 nm) and the emission wavelength was 480 nm (slit width = 5 nm), with a scanning speed of 240 nm/min. Wavelength number was scanned from 190 nm to 270 nm. The fluorescence spectrum of the Th T working solution was deducted as background signal from the fluorescence spectra of the samples. SDS-PAGE was performed for molecular weight estimation of the samples, using a Bio-Rad instrument and precast Mini-protean 4–20% gradient gels. Samples were mixed with stain buffer, using Laemmli mixed with βMe (2-Mercaptoethanol) in 9:1 volume ratio, and then incubated at 90 °C for 10 min and quickly centrifuged for few seconds before starting the measurements. Protein ladder: Bio-Rad precision protein standard. Running buffer: Bio-Rad Tris/Glycine/SDS.

## Supplementary information

Self-assembly of plant protein fibrils interacting with superparamagnetic iron oxide nanoparticles
