# Supplementary material for: Self-assembly of plant protein fibrils interacting with superparamagnetic iron oxide nanoparticles
Source: Sci Rep. 2019 Jun 20;9:8939. doi: 10.1038/s41598-019-45437-z (PMC6586877; doi:10.1038/s41598-019-45437-z)
Supplement: Supplementary file 1 — Self-assembly of plant protein fibrils interacting with superparamagnetic iron oxide nanoparticles [file 41598_2019_45437_MOESM1_ESM.pdf]

# Self-assembly of plant protein fibrils interacting with superparamagnetic iron oxide nanoparticles

Jing Li*, Ievgen Pylypchuk, Daniel Johansson, Vadim G. Kessler, Gulaim A. Seisenbaeva*, Maud Langton*

The Department of Molecular Sciences, SLU - Swedish University of Agricultural Sciences, Box 7015, SE-750 07, Uppsala, Sweden

Correspondence to

Jing Li, email: jing.li@slu.se

Gulaim A. Seisenbaeva, email: gulaim.seisenbaeva@slu.se

Maud Langton, email: maud.langton@slu.se

# Supplementary Information (SI)

## Preparation and characterization of iron oxide ($Fe_3O_4$) superparamagnetic NPs

The NP preparation procedure is reported in detail in our previous work.[1] Synthesis of iron oxide (magnetite) was carried out by co-precipitation of iron salts according to the reaction: $Fe^{2+}$ + $2Fe^{3+}$ + $8NH_4OH \rightarrow Fe_3O_4 + 4H_2O + 8NH_4^+$. In brief, 24 g of ferrous chloride ($FeCl_2$) and 48 g of ferric chloride solution ($FeCl_3$) were dissolved in 1.0 L deionized water. This solution was added dropwise to 250 mL of ammonia solution ($NH_4OH$, 25% in water). Black precipitate (magnetite) was collected and washed several times by distilled water to pH=7. The magnetite was dried overnight at 60°C under nitrogen atmosphere and the powder obtained was stabilized by adding sodium oleate in aqueous solution. Distribution of the dispersion of NPs was confirmed using a transmission electron microscope (TEM) (JEOL 2010). The morphology of the magnetite NPs was characterized using a scanning electron microscope (SEM) (LEO 1430VP, Carl Zeiss, Germany). The NP size distribution was measured by dynamic light scattering (DLS) using a Malvern Zeta-sizer instrument, which was also used for measuring zeta potential. Fourier transform infrared spectroscopy (FTIR) on the fibril and NP-fibril hybrid samples was performed with a Perkin Elmer (model 1720H) spectrometer. Measurements were performed on freeze-dried protein/NP powder at 4000-400 $cm^{-1}$ using the KBr pellet method. Transmittance spectra of KBr were collected as background.

## Protein extraction at high pH

Faba beans (*Vicia faba*, cv. Gloria, provided by RISE, Sweden) were dehulled and milled. Protein extraction was then performed as described in detail elsewhere [2], with the modification that alkaline extraction was carried out at pH 9.0, and subsequent precipitation at pH 4.0. The protein powder was suspended in deionized water at concentrations of 10 mg/mL and 5 mg/mL (dry matter basis). The pH of the protein solutions was adjusted to 2.0 under continuous stirring for 90-120 mins, followed by 30 min of centrifugation and then passage through 0.45 µm nylon syringe filter (Cronus, SMI-LabHut Ltd, USA). After filtration, the pH was re-adjusted to 2.0 if necessary.

**In situ fibrillation of protein and NPs at low pH**

The pH of NP suspensions was adjusted to 2.0 using 6.0 M HCl. The solutions were then incubated in a water bath at 85°C with continuous stirring for 24 h, after which samples were extracted.

**TEM, SEM, DLS and zeta-potential results of iron oxide NPs, fibrils, and NP-fibril hybrids.**

The two components, protein fibrils and NPs, were first characterized separately before forming the hybrid material. The iron oxide (magnetite) was in a superparamagnetic state, as characterized by zero-residual magnetization. TEM (Figure. S1 (a)) shows that each particle can be considered single domain and interparticle magnetic interaction can be neglected. The average size of NPs was ~15 nm, which is in agreement with SEM results (Figure. S1 (b)). Ultra-fine NPs were uniformly distributed without showing any obvious aggregation, indicating that adsorption of oleate improved their stability in suspension. The magnetite NPs were used for subsequent modification of plant (faba bean) protein. The size distribution of NPs was obtained using DLS, providing an average diameter of ~ 42.5 nm (See Figure. S2) The difference in size compared with that measured via TEM and SEM can be explained by the measurement conditions for the different techniques, which can affect the extent of aggregation. The zeta potential of iron oxide NPs was ~-24.6 mV ((Figure. S2), while that of the protein fibrils and the hybrids was 37.8 mV and 42.6 mV, respectively. Generally, NPs with zeta potential values greater than 25 mV or less than -25 mV form stable suspensions. As can be seen, transmission electron microscopy (TEM), scanning electron microscopy (SEM), and dynamic light scattering (DLS) analyses of the NPs revealed uniform dispersion and high stability of the as-prepared NPs, indicating that the oleate modifier successfully stabilizes the NPs. Figure. S3 shows representative Thioflavin T (Th T) fluorescence spectra of another batch of protein and nanoparticle (NP)-fibril hybrid samples.

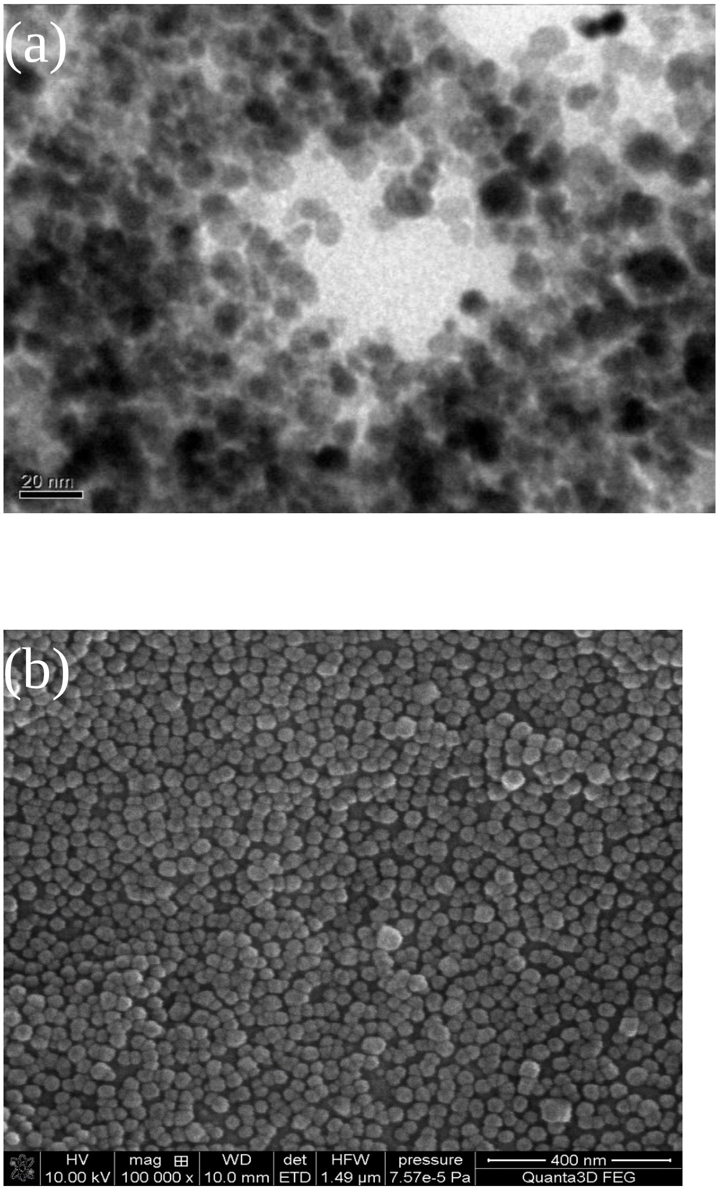


**Figure. S1.** (a) TEM and (b) SEM images of nanosized magnetite ($Fe_3O_4$).

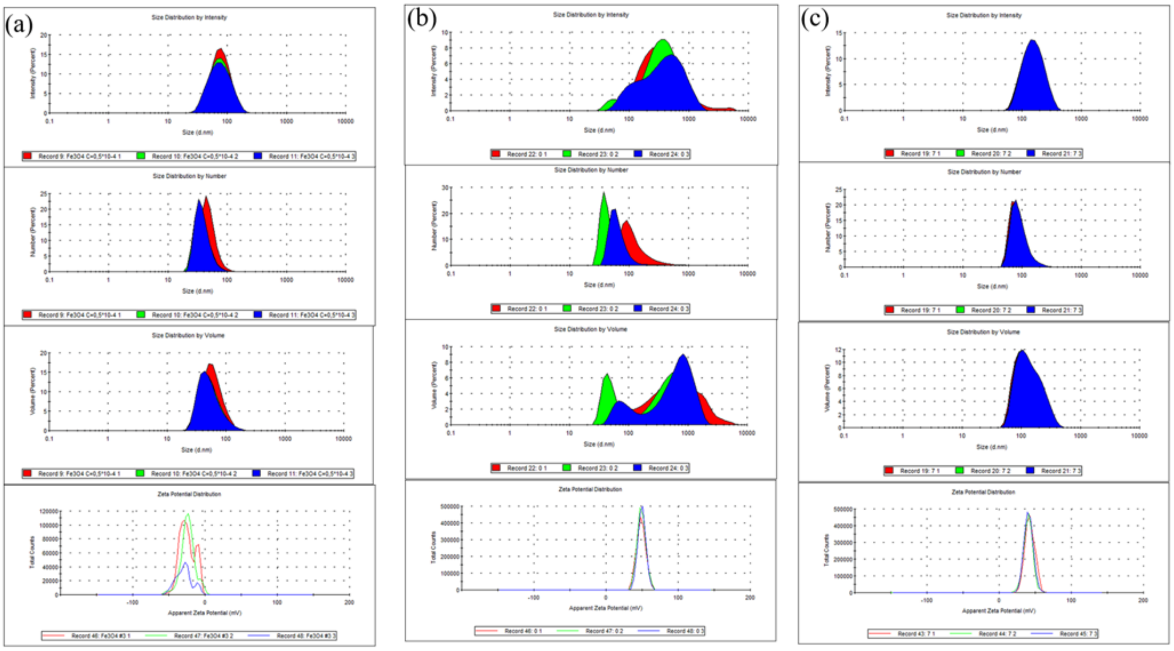


**Figure. S2.** Representative results of size distribution and zeta potential of (a) NPs, and of hybrid samples with (b) 0.03 wt.-% NP and (c) 0.3 wt.-% NP.

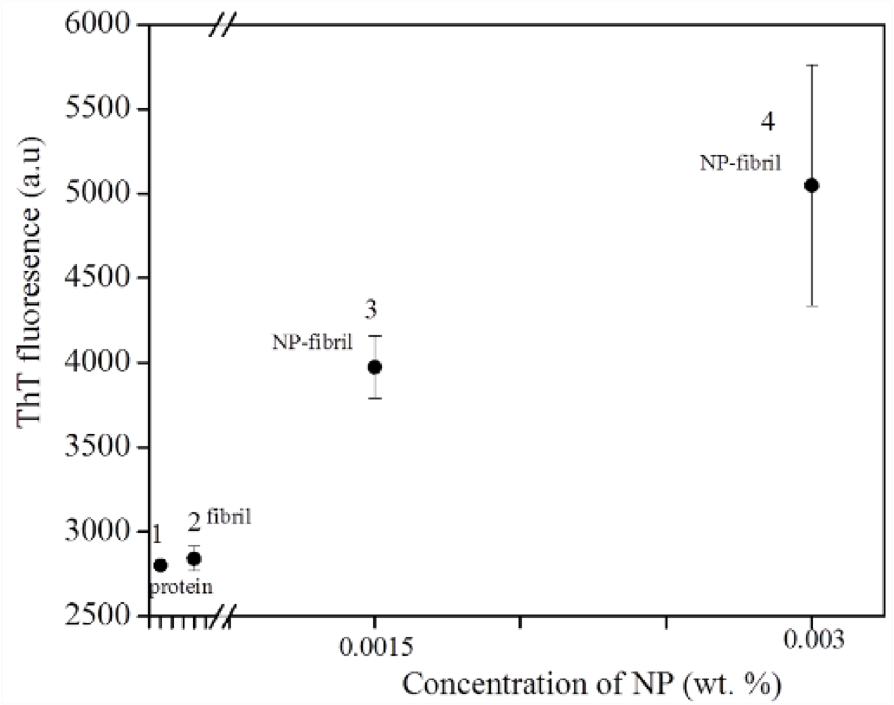


**Figure. S3.** Representative Thioflavin T (Th T) fluorescence spectra of another batch of protein and nanoparticle (NP)-fibril hybrid samples (dot 1: protein; dot 2: fibril; dot 3: 0.0015 wt.-% NPs; dot 4: 0.003. wt. -% NPs) in acidic solution.

**Reference**

1. Gorbyk, P. P., Lerman, L. B., Petranovska, A. L., Turanska, S. P. & Pylypchuk, I. V. Magnetosensitive nanocomposites with hierarchical nanoarchitecture as biomedical nanorobots. in *Fabrication and Self-Assembly of Nanobiomaterials* 289–334 (Elsevier, 2016). doi:10.1016/B978-0-323-41533-0.00010-6

2. Damodaran, S. & K. L.Parkin. *Fennemas's Food Chemistry. Fifth Edition.* (CRC Press, Taylor & Francis Group, 2017).
